# Doping-induced carrier profiles in organic semiconductors determined from capacitive extraction-current transients

**DOI:** 10.1038/s41598-017-05499-3

**Published:** 2017-07-14

**Authors:** Mathias Nyman, Oskar J. Sandberg, Staffan Dahlström, Donato Spoltore, Christian Körner, Yadong Zhang, Stephen Barlow, Seth R. Marder, Karl Leo, Koen Vandewal, Ronald Österbacka

**Affiliations:** 1 Physics/Faculty of Science and Engineering, and Center for Functional Materials, Åbo Akademi University, Porthansgatan 3, 20500 Turku, Finland; 2 Dresden Integrated Center for Applied Physics and Photonic Materials (IAPP) and Institute für Angewandte Physik, Technische Universität Dresden, Nöthnitzer Straße 61, 01187 Dresden, Germany; 3 School of Chemistry and Biochemistry and Center for Organic Photonics and Electronics, Georgia Institute of Technology, Atlanta, Georgia 30332-0400 United States

## Abstract

A method to determine the doping induced charge carrier profiles in lightly and moderately doped organic semiconductor thin films is presented. The theory of the method of Charge Extraction by a Linearly Increasing Voltage technique in the doping-induced capacitive regime (doping-CELIV) is extended to the case with non-uniform doping profiles and the analytical description is verified with drift-diffusion simulations. The method is demonstrated experimentally on evaporated organic small-molecule thin films with a controlled doping profile, and solution-processed thin films where the non-uniform doping profile is unintentional, probably induced during the deposition process, and a priori unknown. Furthermore, the method offers a possibility of directly probing charge-density distributions at interfaces between highly doped and lightly doped or undoped layers.

## Introduction

Organic semiconducting materials hold great potential for use in future electronic devices such as solar cells, transistors, and thermoelectric generators. Contrary to their inorganic counterparts, they are typically used in their intrinsic, i.e. (at least nominally) undoped form when used as active layers. However, doped organic semiconductors, both vacuum evaporated and solution processed, are becoming increasingly common as contacts and contact interlayers, and are already commercially used in organic light-emitting diodes (OLEDs)^1–5^. Doping controls the Fermi level and thus allows to increase the charge-carrier density and thereby the conductivity. Ohmic and highly conductive contacts are essential in organic electronic devices, as imperfect contacts considerably impair performance^6–9^. In order to utilize doping as a mechanism for controlling the work function and conductivity of doped layers, a sound understanding of the doping mechanisms in organic materials is needed.

The doping processes in organic materials are more complex than in inorganic materials due to their disordered nature, weak intermolecular coupling, and low dielectric screening. In fact, the underlying mechanisms are still not well understood^9^. The doping efficiency (defined as the ratio of free charge carriers contributing to conductivity, to the number of dopants), for example, is often considerably lower in organic materials; the reasons are still being investigated and several mechanisms have been proposed^10–12^. This highlights the importance of being able to accurately measure the free-carrier concentration, preferably with in-device techniques. Furthermore, in order to use highly doped layers as contacts and contact interlayers it is important to make sure that the dopants do not diffuse from the doped layer into the intrinsic layer, as this will be detrimental for device performance. Recently it has been shown that, in some cases, dopants can diffuse several hundreds of nanometers from the contacts into the active layer, resulting in lower concentration of free carriers in the doped layer and the formation of an unwanted depletion layer in the “intrinsic” layer^13, 14^. In addition, organic materials are rarely truly intrinsic due to their sensitivity to oxygen and, at least relative to crystalline inorganic semiconductors, high levels of impurities remaining from their synthesis^15–17^.

The concentration of free charges, *N*_*free*_, in a doped semiconductor layer is related to the depletion-region width *w* and potential drop *U* as ${N}_{free}\,=2{\epsilon }{{\epsilon }}_{0}U/(e{w}^{2})$, where ${\epsilon }$ is the relative dielectric constant, ${{\epsilon }}_{0}$ is the vacuum permittivity and *e* is the elementary charge^9^. Common techniques to measure the concentration of free carriers includes ultraviolet photoelectron spectroscopy (UPS) and impedance spectroscopy, while recently a way of determining the free-carrier concentration from conductivity and Seebeck measurements was presented^12, 18–21^. However, UPS is only sensitive to the surface, and furthermore requires advanced equipment and is time consuming. The Seebeck and conductivity measurements, as well as impedance spectroscopy, are in-device measurements, however; Seebeck and conductivity measurements require additional measurements or assumptions of the carrier mobility^20, 21^. Impedance spectroscopy typically requires equivalent-circuit modeling and careful analysis of the data.

**Figure 1:**
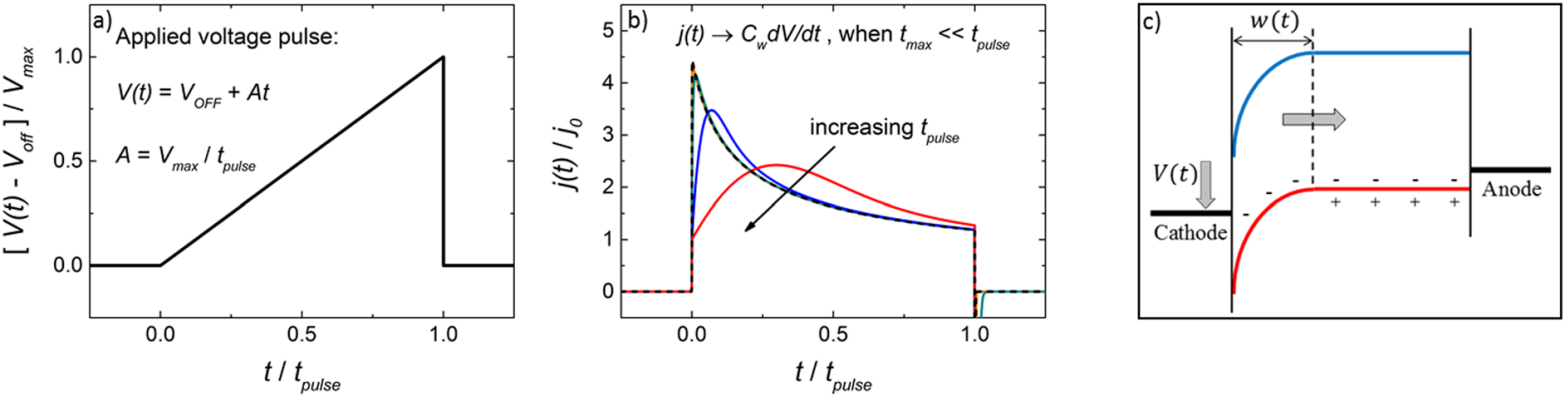
(**a**) A linearly increasing voltage pulse, *V*(*t*) = *V*_*OFF*_ + *At*, is applied in reverse bias to extract charge carriers at an applied dc voltage *V*_*OFF*_. (**b**) The corresponding current transients *j(t)* (colored solid lines) are determined by the rate of charge extraction, where the time of the current maximum, *t*_*max*_, depends on the mobility of the extracted carriers and ${j}_{0}={C}_{geo}A$ is the current response due to the geometric capacitance (*C*_*geo*_). At slow voltage pulses $\,{t}_{pulse}\gg {t}_{max}$ the normalized current transient saturate as $j(t)/{j}_{0}\to {C}_{w}/{C}_{geo}$ (dashed line), where *C*_*w*_ is the depletion layer capacitance. Note that for an accurate evaluation of *j*(*t*) at slow pulses, the current transients typically have to be corrected for (time-independent) parasitic leakage currents. (**c**) Energy-band diagram at slow voltage pulses where free charges have had time to equilibrate and a depletion region is formed at the cathode, at *x* = 0, in the case of p-doping. Upon an applied voltage pulse holes are extracted (in the case of p-doping) and the depletion region width, *w*, increases towards the anode at *x* = *d*.

We recently demonstrated that the Charge Extraction by a Linearly Increasing Voltage technique in the doping-induced capacitive regime (doping-CELIV) can be used to determine the built-in voltage and carrier concentration in sandwich-type diode devices^22^. In a uniformly p-doped device a depletion region is formed at the cathode with width *w*_*0*_ (at an applied steady-state voltage *V*_*OFF*_) given by:

1 $${w}_{0}=\sqrt{\frac{2{\epsilon }{{\epsilon }}_{0}({V}_{bi}+{V}_{OFF})}{ep}}$$

where *V*_*bi*_ is the built-in potential and *p* is the concentration of holes. By applying a linearly increasing voltage pulse with slope *A* = *V*_*max*_/*t*_*pulse*_, where *V*_*max*_ is the amplitude and *t*_*pulse*_ the length of the voltage pulse, in reverse direction of the diode, the doping-induced charge carriers can be extracted. A schematic picture is shown in Fig. 1. In the doping-induced capacitive regime, the corresponding extraction current transient *j*(*t*) is given by:

2 $$j(t)={C}_{w}\frac{dV}{dt}$$

where ${C}_{w}={\epsilon }{{\epsilon }}_{0}/w\,\,$is the depletion layer capacitance and

3 $$w(t)=\sqrt{\frac{2{\epsilon }{{\epsilon }}_{0}}{ep}[V(t)+{V}_{bi}]}$$

where *V*(*t*) = *At* + *V*_*OFF*_ is the total applied voltage over the device. The requirement for being in the doping-induced capacitive regime is that *w* < *d*, where d is the active layer thickness, and *t*_*pulse*_ ≫ *t*_*max*_, where *t*_*max*_ is the time the current transient reaches its maximum value.

The method is fast and simple and, similarly to impedance spectroscopy, relies on Mott-Schottky analysis of the capacitance. The advantage of the doping-CELIV method is that it allows for direct determination of both the mobility and the carrier density without the need for equivalent circuit modeling. The method has been successfully used to identify a cause of unintentional doping in polymer-fullerene blends and clarify the light-soaking effect in inverted solar cells with a titanium-oxide-modified cathode^14, 23^. The doping-CELIV theory presented by Sandberg *et al*.^22^, however, is only valid for uniform carrier profiles, which is a severe limitation.

In this paper, we extend the theory of the doping-CELIV method to the case of non-uniform doping-induced carrier profiles. Furthermore, a method of determining the charge-carrier profile from the extraction current transient is shown. An expression for the capacitive extraction current is derived analytically for a step-wise and a linear doping profile; the theory is confirmed with numerical drift-diffusion simulations. The method is furthermore demonstrated experimentally on vacuum-evaporated thin films with a controlled doping profile, and solution-processed films where the non-uniformity of the doping profile is unintentional and most likely a consequence of the morphology.

## Results and Discussion

### Analytical description of the model

Consider a p-doped device of thickness *d*, consisting of a depleted space-charge region adjacent to the cathode (*x* = 0), where *w* < *d* is the thickness of the depletion region. Assuming a one-dimensional, planar current flow, the total transient current density *j*(*t*) is given by^24^

4 $$j(t)={J}_{c}(x,t)+{\epsilon }{{\epsilon }}_{0}\frac{\partial E(x,t)}{\partial t}$$

where *J*_*c*_(*x,t*) is the conduction current and ${\epsilon }{{\epsilon }}_{0}\frac{\partial E(x,t)}{\partial t}$ is the displacement current. Note that the terms *J*_*c*_(*x,t*) and ${\epsilon }{{\epsilon }}_{0}\partial E(x,t)/\partial t$ can individually depend on *x*; however, their sum *j*(*t*) must be *independent* of *x*, as required by the continuity equation *dj*/*dx* = 0. Here *E*(*x,t*) is the electric field, related to the applied transient voltage *V*(*t*) via

5 $$V(t)+{V}_{bi}={\int }_{0}^{d}E(x,t)dx$$

In the following we assume that $V(t)+{V}_{bi}\gg kT/e$, where *k* is Boltzmanns constant and *T* is the temperature. Applying the voltage pulse in reverse bias (or with a blocking cathode), so that charge carriers are extracted at the anode, the transient conduction current *J*_*c*_(*x*, *t*) will be vanishingly small in the depleted region. Assuming that all charge carriers within the depletion region are extracted, the Poisson equation reads

6 $$\frac{{\rm{\partial }}E(x,t)}{{\rm{\partial }}x}=\{\begin{array}{c}-\frac{e}{\epsilon {\epsilon }_{0}}{N}_{p}(x),\,0 < x < w(t)\\ \frac{e}{\epsilon {\epsilon }_{0}}[p(x,t)-{N}_{p}(x)],\,w(t)\le x < d\end{array}$$

for the case with a free charge-carrier density *p*(*x*, *t*) and an arbitrary density profile *N*_*p*_(*x*) of negatively charged (ionized) p-dopants (assumed to be fixed).

During sufficiently slow ramp-up voltage pulses (*t*_*pulse*_ ≫ *t*_*max*_), the charge carriers maintain equilibrium under steady-state conditions, and *p*(*x*, *t*) will not significantly deviate from its quasi-equilibrium density *p*(*x*)^22^. Under these conditions, the electric field at the anode becomes time-independent *E*(*d,t*) = *E*_*an*_ (assuming *w* < *d*); integrating Eq. (6) then yields

7 $$E(0,t)={E}_{an}+{\int }_{0}^{w(t)}\frac{e{N}_{p}(x)}{{\epsilon }{{\epsilon }}_{0}}dx-{\int }_{w(t)}^{d}\frac{e[p(x)-{N}_{p}(x)]}{{\epsilon }{{\epsilon }}_{0}}dx$$

Furthermore, after integrating Eq. (5) by parts we find

8 $$V(t)+{V}_{bi}-{E}_{an}d=\,-{\int }_{0}^{d}x\frac{dE}{dx}dx={\int }_{0}^{w(t)}\frac{e{N}_{p}(x)x}{{\epsilon }{{\epsilon }}_{0}}dx-{\int }_{w(t)}^{d}\frac{e[p(x)-{N}_{p}(x)]x}{{\epsilon }{{\epsilon }}_{0}}dx$$

and concomitantly $dV/dw=ep(w)w/{\epsilon }{{\epsilon }}_{0}$. Hence, utilizing the chain rule $\partial E(0,t)/\partial t=(dE(0,t)/dw)(dw/dt)$, noting that *J*_*c*_(*0,t*) = 0 and $\,dE(0,t)/dw=ep(w)/{\epsilon }{{\epsilon }}_{0}$, the total current density Eq. (4) simplifies to

9 $$j={\epsilon }{{\epsilon }}_{0}\frac{\partial E(0,t)}{\partial t}=ep(w)\frac{dw}{dt}=\frac{{\epsilon }{{\epsilon }}_{0}A}{w}$$

where $A\equiv \frac{dV}{dt}$. As expected, at slow pulses, the transient current density becomes directly proportional to the depletion-layer capacitance $\,{C}_{w}={\epsilon }{{\epsilon }}_{0}/w$, also for the case of an arbitrary carrier profile. Utilizing the last equality in Eq. (9), the charge-carrier density profile *p*(*w*) can be furthermore related to *w* as ${\epsilon }{{\epsilon }}_{0}/ep(w)=wdw/dV=d[{w}^{2}/2]/dV$. Noting that

10 $$w=\frac{\epsilon {\epsilon }_{0}A}{j}={(\frac{j}{{j}_{0}})}^{-1}d$$

it then follows that

11 $$p(w)=\frac{2}{e{\epsilon }{{\epsilon }}_{0}{A}^{2}}{[\frac{d}{dV}{j}^{-2}]}^{-1}=\frac{2{\epsilon }{{\epsilon }}_{0}}{e{d}^{2}}{[\frac{d}{dV}{(\frac{j}{{j}_{0}})}^{-2}]}^{-1}$$

Here, ${j}_{0}\equiv {\epsilon }{{\epsilon }}_{0}A/d$ is the geometric capacitive response, corresponding to a fully depleted (undoped) device. Utilizing Eq. (10) and Eq. (11), the doping-induced carrier profile of the extracted charge carriers can be obtained.

### Drift-diffusion simulations

**Figure 2:**
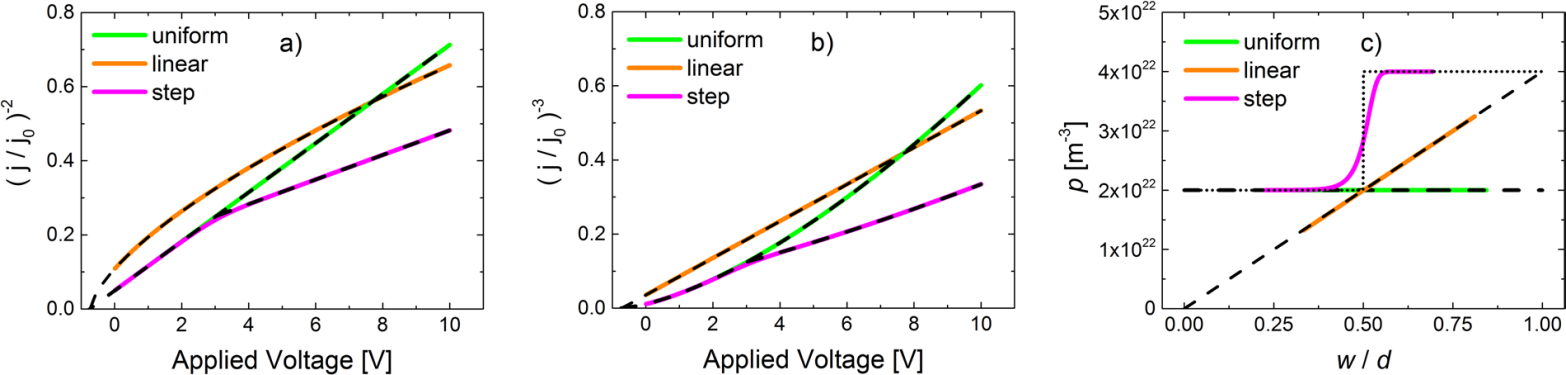
(**a**) Simulated doping-CELIV current transients, plotted as (*j/j*_*0*_)^−2^ vs applied transient voltage, for *V*_*OFF*_ = 0, are depicted by the solid colored lines. The analytical approximations of $\,{(j/{j}_{0})}^{-2}={w}^{2}/{d}^{2}$ assuming $p(x)={N}_{p}(x)$ (Eq. (12) and Eq. (13)) are indicated by dashed lines. (**b**) The same current transients as in a), but plotted as (*j/j*_*0*_)^−3^ instead. (**c**) The carrier density profiles, as extracted from the simulated CELIV current transients in a), using Eq. (11) and Eq. (10) (to obtain *p*(*w*) and *w*, respectively), are depicted by the colored lines. The corresponding densities of ionized dopants, *N*_*p*_(*x*), are depicted by the dashed, short-dashed, and dotted lines for uniform, linear and step profiles, respectively.

In Fig. 2a, simulated CELIV current transients in the capacitive regime are shown. A previously developed macroscopic drift-diffusion model is used for the simulations^7, 22^. The current transients (solid lines), plotted as (*j*/*j*_0_)^−2^ vs *V*(*t*), are simulated for three different ionized dopant profiles, *N*_*p*_(*x*): uniform, linear, and step profile. In accordance with Eq. (10), we should have (*j*/*j*_0_)^−2^ = *w* ^2^/*d* ^2^. Moreover, under the simplifying approximations that *E*_*an*_ = 0 and *p*(*x*) = *N*_*p*_(*x*) for *x* ≥ *w*, analytical expressions for the (quasi-equilibrium) depletion-layer thickness *w* can be directly obtained by solving Eq. (8). For a linearly changing profile, *N*_*p*_(*x*) = *ax*, one finds

12 $$w=\sqrt[3]{\frac{3{\epsilon }{{\epsilon }}_{0}}{ea}[V(t)+{V}_{bi}]}$$

For a step profile, ${N}_{p}(x)={N}_{p1}$ for 0 < *x* < *d*_*1*_ and ${N}_{p}(x)={N}_{p2}$ for *d*_*1*_ < *x* < *d*, we obtain

13a $$w=\sqrt{\frac{2{\epsilon }{{\epsilon }}_{0}}{e{N}_{p1}}[V(t)+{V}_{bi}]},\,\mathrm{when}\,w < {d}_{1}$$

13b $$w=\sqrt{\frac{2{\epsilon }{{\epsilon }}_{0}}{e{N}_{p2}}[V(t)+{V}_{bi}+\frac{e[{N}_{p2}-{N}_{p1}]{d}_{1}^{2}}{2{\epsilon }{{\epsilon }}_{0}}]},\,\mathrm{when}\,w > {d}_{1}$$

where *N*_*p2*_ ≥ *N*_*p1*_. The case of a uniform profile (*N*_*p*_(*x*) = *N*_*p*_) corresponds to the case *N*_*p*_ = *N*_*p2*_ = *N*_*p1*_ in Eq. (13). The analytical approximations are depicted by the black dashed lines in Fig. 2a. Comparing the analytical expressions with the simulated transients in Fig. 2a, nearly perfect agreement is found, confirming that at slow pulses the CELIV current transients are indeed given by Eq. (9). In the uniform and linear case (Eq. (12)), a straight-forward analysis can be performed by plotting 1/*j* ^2^ or 1/*j* ^3^ respectively, as a function of applied voltage, which gives a straight line over the capacitive region of the current transient. From the slope of this line the uniform free carrier concentration, *p* = *N*_*p*_, or the gradient, *a*, of the linear profile can be calculated. The (*j*/*j*_0_)^−3^ vs *V*(*t*) plot of the current transients in Fig. 2a are shown in Fig. 2b.

In Fig. 2c the spatial profiles of the extracted carrier density *p*(*w*) vs *w/d*, obtained using Eq. (10) and Eq. (11), of the simulated transients are depicted, indicated by the colored solid lines. The corresponding *N*_*p*_(*w*) are indicated by the dashed and dotted black lines. Indeed, for the uniform and the linear doping profiles, the extracted carrier profiles coincide well with the fixed dopant profile. This also justifies the approximation that *p*(*x*) *≈ N*_*p*_(*x*) when *x* ≥ *w*, assumed in Eq. (3) and Eq. (12). For the step profile, however, we see that a deviation between the extracted *p*(*w*) and *N*_*p*_(*w*) is found when *w* is close to *d*_*1*_, where *N*_*p*_ is changed abruptly.

**Figure 3:**
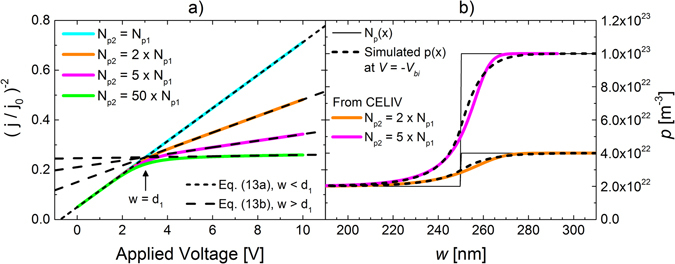
(**a**) Simulated capacitive CELIV current transients for a device with a step profile with different steps where ${N}_{p}(x)={N}_{p1}$ for 0 < *x* < *d*_*1*_, ${N}_{p}(x)={N}_{p2}$ for *d*_*1*_ < *x* < *d*, where *d*_*1*_ = 250 nm and *N*_*p1*_ = 2 × 10^22^ m^−3^. (**b**) The carrier-density profiles as extracted from the CELIV current transients, using Eq. (11) and Eq. (10), are depicted by the colored lines. For comparison, the actual quasi-equilibrium hole densities *p*(*x*), simulated at *V* = *−V*_*bi*_, are depicted by the dashed lines; the corresponding input concentration of ionized dopants *N*_*p*_(*x*) is indicated by the thin black lines.

To elucidate this behavior, the simulated current transients for the case with a step profile with different *N*_*p2*_, keeping *N*_*p1*_ = 2 × 10^22^ m^−3^ fixed, are shown in Fig. 3a. It is seen from Fig. 3a that the deviation (from Eq. (13)), which occurs when *w* is close to *d*_*1*_, becomes more visible at larger dopant concentrations *N*_*p2*_. This is simply due to the fact that at larger carrier density, a larger voltage *V* is required to increase the depletion region (since $\,d{w}^{2}/dV\propto 1/p$). To determine the origin of the deviation, the actual quasi-equilibrium hole densities *p*(*x*), evaluated at *V* = −*V*_*bi*_ and depicted for *N*_*p2*_ = 2*N*_*p1*_ and *N*_*p2*_ = 10*N*_*p1*_, are simulated in Fig. 3b. It is seen that a significant portion of the free carriers have diffused from the high-dopant region to the low-dopant region due to the abrupt change in the doping profile, leading to *p*(*x*) *≠ N*_*p*_(*x*) in the region close to *x* = *d*_*1*_. The characteristic decay length of this carrier diffusion is given by the Debye length $\,{L}_{D}=\sqrt{{\epsilon }{{\epsilon }}_{0}kT/({e}^{2}p)}\,$ ^25, 26^; here, a free-carrier concentration of $p\,=\,\,$2 × 10^22^ m^−3^ corresponds to *L*_*D*_ ~ 15 nm. When comparing the carrier-density profile *p*(*w*) of extracted carrier density, as obtained from the current transients, with the actual quasi-equilibrium hole density *p*(*x*), a good agreement is indeed found in Fig. 3b. We note that small deviations are, however, present close to *x* = *d*_*1*_. These deviations are expected due to the depletion-edge approximation made in Eq. (6). While the depletion-edge approximation provides accurate carrier profiles for smooth profiles (such as the uniform and the linear one), deviations are obtained for highly non-uniform profiles^26, 27^. As these deviations are relatively small, however, Eq. (9) still provides a good approximation of the extracted carrier profile for steep profiles.

### Experimental determination of doping profiles

**Figure 4:**
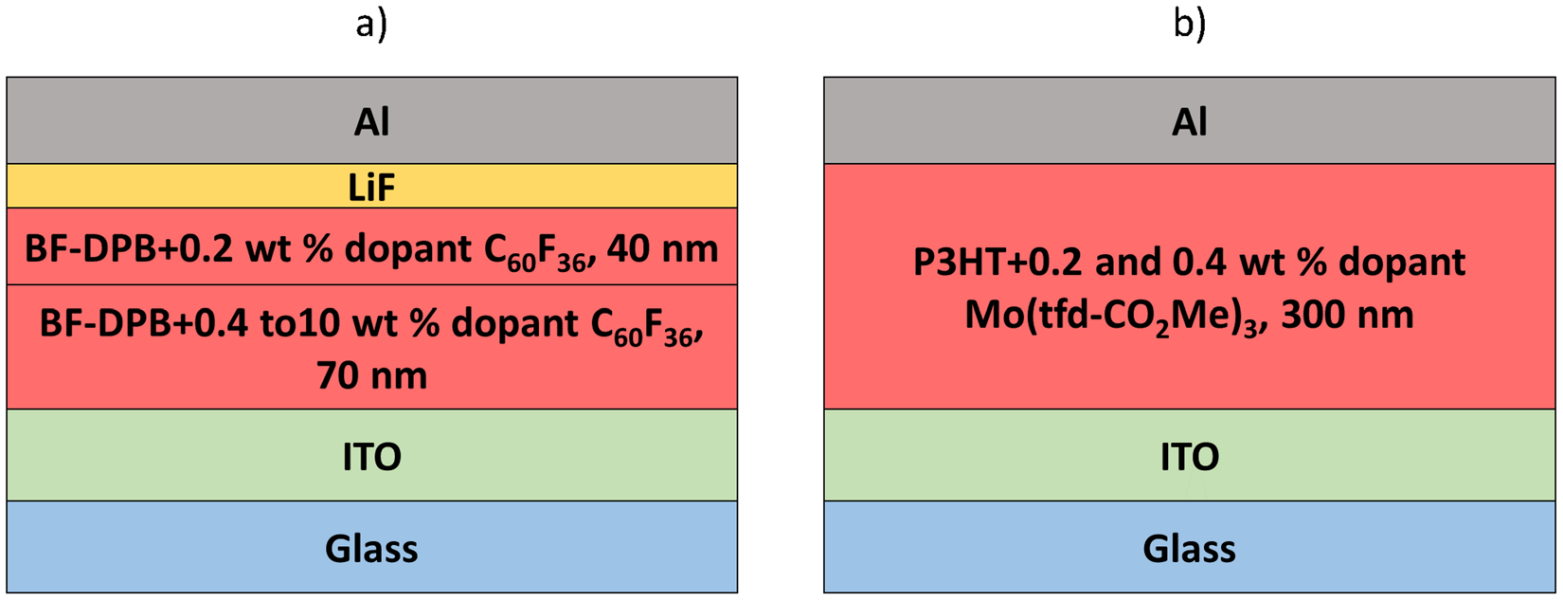
Schematic picture of the device structure of (**a**) vacuum-evaporated thin film devices with a BF-DPB active layer doped with C_60_F_36_, forming a step wise distribution, and (**b**) solution-processed polymer thin film devices with a P3HT active layer doped with Mo(tfd-CO_2_Me)_3_.

The doping-CELIV profile method is demonstrated experimentally on vacuum-evaporated thin films with a controlled, step-wise distribution of dopants, and on solution-processed polymer thin films where any profile is unintentional. In the vacuum-evaporated thin films *N,N*ʹ-Bis(9,9-dimethyl-fluoren-2-yl)-*N,N*ʹ-diphenyl-benzidine (BF-DPB) is used as the host material and C_60_F_36_ is used as dopant, these materials have been previously used in thermoelectric devices and in hole-transporting layers for OLEDs^21, 28^. The devices consist of a 70 nm thick layer with varying dopant to host weight ratios (0.4 to 10 weight %) and a 40 nm thick lightly doped layer (0.2 weight %) on top; ITO and LiF/Al are used as bottom anode and top cathode, respectively, see Fig. 4a for a schematic of the device structure. In the solution-processed thin films a 300 nm thick layer of the conjugated polymer poly(3-hexylthiophene) (P3HT) doped with the soluble p-dopant molybdenum tris[1-(methoxycarbonyl)−2-(trifluoromethyl)-ethane-1,2-dithiolene] (Mo(tfd-CO_2_Me)_3_) has been tested. Devices with ITO as the bottom anode and Al as the top cathode were made. The device structure is shown in Fig. 4b. To check that the applied voltage pulses are slow enough we performed measurements with varying *A* and plotted the extraction current normalized to *j*_*0*_ as function of voltage (see Supplementary Information Figure S1). The transients overlap when the pulses are slow enough. Furthermore we performed measurements on undoped samples to show that the results for the doped samples are not affected by band-bending (see Supplementary Information Figure S2).

**Figure 5:**
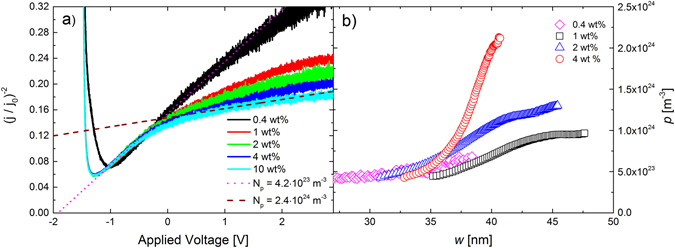
(**a**) Capacitive extraction current transients of BF-DPB:C_60_F_36_ devices with two layers of different dopant concentrations; in the lower doped layer (*d* = 40 nm) the C_60_F_36_ to BF-DPB weight ratio is 0.2% (corresponding to molar ratio of 0.001) in the more heavily doped layer (*d* = 70 nm) the dopant concentration is varied from 0.4 wt% to 10 wt% (corresponding to molar ratios of 0.002 and 0.057). In (**b**) the charge-carrier profiles (as determined by Eq. (11)) of four devices with different dopant concentration in the highly doped layer are shown.

Figure 5a shows the inverse square of the extraction currents for vacuum evaporated thin film devices with different dopant concentrations in the highly doped layer. A clear “knee” can be seen at around −0.3 V in the plots for all devices, corresponding to the depletion region crossing over from the lower doped to the higher doped layer. For voltages below −0.3 V the slopes are more-or-less the same in all devices; the magenta dotted line is a guide to the eye corresponding to a free-carrier concentration of 4.2 × 10^23^ m^−3^. This would correspond to a doping efficiency of 42% in the lightly doped layer. The slopes for voltages more positive than −0.3 V are seen to decrease for increasing dopant concentrations, as expected, the wine-colored dashed line is a guide to the eye corresponding to a free-carrier concentration of 2.4 × 10^24^ m^−3^. We note that the charge-carrier mobility in this system is rather low, and appears to be highly dispersive, which results in a broad transport-dominated region in the beginning of the extraction-current transient.

In Fig. 5b the free-carrier concentrations (determined from Eq. (11)) as a function of *w* (determined from Eq. (10)) of devices with four different dopant concentrations in the highly doped layer, are shown. In the 1 wt % case it can be seen that the free-carrier concentration starts to increase when *w* > 35 nm, going from 5 × 10^23^ m^−3^ and appears to saturate at ca. 10^24^ m^−3^ for *w* = 45 nm. The reason why the transition occurs over ~ 10 nm is partly due to the fact that the interface between the two layers is not completely flat, but the dominant reason is the diffusion of free holes from the heavily doped to the lightly doped layer, as seen also in Fig. 3. The doping efficiency in the 1 wt % layer can then be estimated to 19%, which is slightly lower than in the 0.2 wt % case. For comparison, the free-carrier concentration in the 0.4 wt % layer is 6 × 10^23^ m^−3^ (as estimated from Figure 5b) corresponding to a doping efficiency of 29%. In the 2 and 4 wt% cases, no clear saturation is seen in Fig. 5b; the free-carrier concentration and thus the doping efficiency cannot be reliably estimated. The free-carrier concentration in the low doped layer is estimated at 4.2 × 10^23^ m^−3^ from Fig. 5b (for *w* < 30 nm). The measured free-carrier concentrations and doping efficiencies (where available) as well as the molecular ratios and concentration of dopants for all the different vacuum-evaporated layers are shown in Table 1.

**Table 1:** The dopant to host weight % (wt %), dopant concentration, measured free-carrier concentration, and doping efficiency of the doped layers used.

Dopant to host wt %	Dopant conc. [m^−3^]	Free carrier conc. [m^−3^]	Doping efficiency [%]
0.2	1 × 10^24^	4.2 × 10^23^	42%
0.4	2.1 × 10^24^	6.0 × 10^23^	29%
1.0	5.2 × 10^24^	1.0 × 10^24^	19%
2.0	1.1 × 10^25^	N/A	N/A
4.0	2.1 × 10^25^	N/A	N/A
10.0	5.7 × 10^25^	N/A	N/A

Experimentally it is challenging, or impossible, to obtain the full carrier profile of the organic semiconductor layer stack. Due to charge injection at large offset voltages and limitations due to charge transport, it is not possible to obtain a capacitive extraction current for very small depletion-region widths. Furthermore, at large applied extraction voltages the conduction term in Eq. (4) will become significant which renders the profile method unreliable. In Fig. 5 the layer thicknesses and experimental parameters are chosen so that the measurement will be most reliable when the depletion region crosses over from the lightly doped to the heavily doped layer. However, it is possible that the free-carrier concentration in the lightly doped layer is overestimated and correspondingly underestimated in the heavily doped layer due to diffusion of free holes. However, in general, the measured doping profiles correspond rather well with the a priori known dopant profile, which gives confidence in the method.

**Figure 6:**
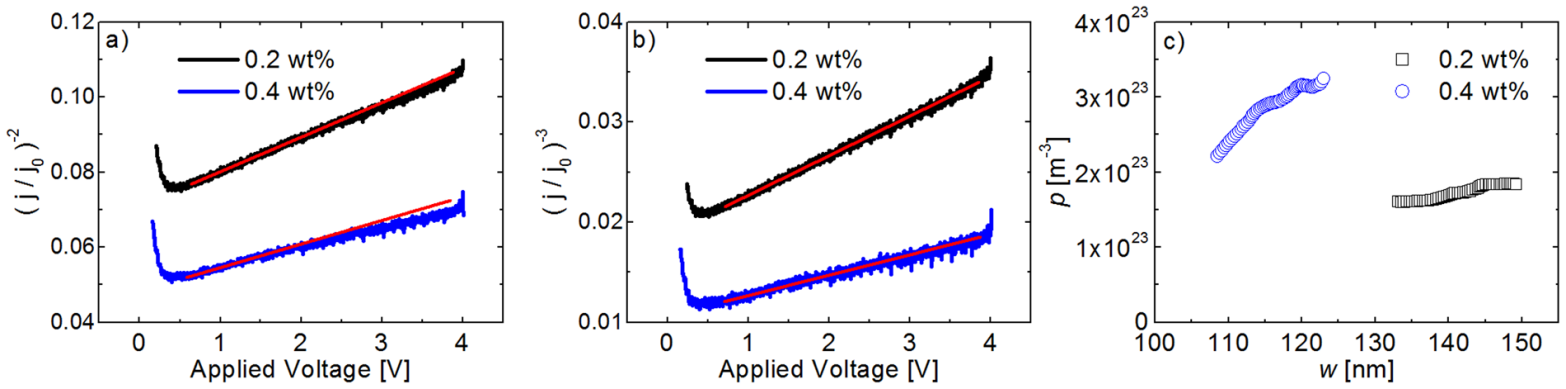
Capacitive extraction-current transients of P3HT devices is plotted as (**a**) (*j*/*j*_*0*_)^−2^ and (**b**) (*j*/*j*_*0*_)^−3^ vs applied voltage, for 0.2 wt % and 0.4 wt % dopants, where d = 300 nm. In this case the carrier profile is linearly increasing and a linear dependence can be seen in (**b**) in accordance with Eq. (12). The red lines in (**a** and **b**) are guides to the eye representing a straight line. In (**c**) the corresponding charge carrier profile is shown as a function of the depletion layer width.

In the following section we apply the doping-profile method to solution-processed thin film devices. The ITO/P3HT/Al device structure is illustrated in Fig. 4b. Layers of P3HT doped with Mo(tfd-CO_2_Me)_3_ have previously been demonstrated to function as hole-transport layers, where the doped layers were characterized by UPS and conductivity measurements^5^. Extensive diffusion of this dopant between doped and undoped layers of P3HT has been identified via secondary ion mass spectrometry^13^. In our case a single doped layer is measured in order to demonstrate the usefulness of the presented method for achieving the doping-induced carrier profile. The dopant has been mixed in solution with P3HT prior to spin-coating a 300 nm thick active layer. Current transients of devices with 0.2 wt % and 0.4 wt % dopants is plotted as (*j*/*j*_*0*_)^−2^ and (*j*/*j*_*0*_)^−3^ as a function of the applied voltage in Fig. 6a and b respectively. The measured carrier profiles are plotted in Fig. 6c and are increasing towards the bottom anode both for 0.2 wt % and 0.4 wt %. The profile can be approximated as linearly increasing in the measured region and in Fig. 6b a linear dependence of (*j*/*j*_*0*_)^−3^ vs applied voltage can be seen as predicted for a linear profile by Eq. (12). This unintentionally non-uniform profile is most likely an effect originating from the active-layer morphology. Assuming the same profile for higher concentrations when used as a hole-transport layer, the consequences are not necessarily detrimental. By making a linear fit to the carrier profiles a slope of 2.1 × 10^21^ m^−3^nm^−1^ and 6.5 × 10^21^ m^−3^nm^−1^ for 0.2 wt % and 0.4 wt % respectively is obtained. For thin hole-transport layers this kind of moderately non-uniform carrier profile is unlikely to affect the contact properties negatively.

Assuming that the dopant molecules follow the same linear distribution as the measured free-carrier concentration throughout the active layer, doping efficiency can be estimated to 10.7% and 14.0% for 0.2 wt % and 0.4 wt % dopants respectively. These doping efficiencies can only be considered as indicative of the actual values at these concentrations; since the distribution of free charge carriers is measured only in roughly 20 nm of the 300 nm thick active layer, assumptions of the complete profile is challenging to make.

In all the devices measured here p-dopants have been used, therefore it is clear that the depletion region will be adjacent to the cathode. If the method is applied to a device where the origin of the doping is unknown, it is not necessarily possible to say whether the depletion region will be at the anode (n-doping) or the cathode (p-doping). This is particularly the case in donor-acceptor blends, where p-doping (of the donor) is as likely to occur as n-doping (of the acceptor). However, we argue that obtaining the shape of the doping profile can be a valuable tool in trying to determine the source of the doping.

## Conclusions

We have presented an in-device method to measure arbitrary doping-induced charge-carrier profiles in low and moderately doped semiconductors by extending the doping-CELIV theory. In addition to the analytical description for extracting arbitrary charge-carrier profiles, the capacitive extraction currents for step and linear profiles have been derived, and the theory has been verified by drift-diffusion simulations. Furthermore, the method has been demonstrated experimentally on organic small-molecule thin films with controlled concentrations of dopants following a step profile, and on solution-processed thin films where an unintentional doping profile, with carrier concentration linearly increasing towards the bottom anode, can be seen in the measured region of the active layer. The method can be used to determine arbitrary charge carrier profiles of lightly and moderately doped semiconductor thin films, also in bulk heterojunctions. However, due to diffusion of charges from highly doped to lightly doped regions, abrupt changes in the doping profile will be smoothed out in the profile obtained by the measurement.

## Experimental Section

Small molecules thin films devices were thermally evaporated at ultra-high vacuum (base pressure < 10^−7^ mbar) onto a glass substrate with a pre-structured ITO contact (Thin Film Devices, USA). The active layer comprises 70 nm of N,N’-bis(9,9-dimethyl-fluoren-2-yl)-N,N’-diphenyl-benzidine (BF-DPB) (Synthon Chemicals GmbH, Germany) as host material doped with C_60_F_36_ (Ionic Liquids Technologie GmbH, Germany) in varying dopant to host weight ratios (0.4 to 10 weight %). Afterwards, a 40 nm thick lightly doped layer (0.2 weight %) was evaporated. The devices are completed with 2 nm LiF and 100 nm Al as top cathode. The devices are defined by the geometrical overlap of the bottom and the top contact with an active area of 6.44 mm^2^. To avoid exposure to ambient conditions, the organic part of the device is covered by a small glass substrate, glued on top.

Solution-processed samples were prepared using ITO covered borosilicate glass (from Präzisions Glas & Optik GmbH) as substrate. Half of the substrate was etched with concentrated aqueous HCl (37–38%) for roughly 40 min. The substrates were subsequently cleaned in a 1:1:5 blend of H2O2, NH3, and water in an ultrasonicator at 70 °C for 30 min. Solutions of the p-dopant, Mo(tfd-CO_2_Me)_3_^5^, and P3HT (from Sigma-Aldrich) were prepared in chlorobenzene and mixed at 0.2 and 0.4 weight % dopants. The 300 nm thick P3HT films were spin-cast from a doped 25 mg/ml solution. The films were annealed at 120 °C for 10 min. A 60 nm thick layer of Al was thermally evaporated as a top cathode with approximately 4 mm^2^ overlap with the ITO bottom anode. Spin-coating, annealing and thermal evaporation was carried out in a nitrogen glovebox. The active layer thicknesses were determined using atomic force microscopy. CELIV measurements were performed as reported earlier^22, 23^.

### Data Availability

The datasets generated during and/or analysed during the current study are available from the corresponding author on reasonable request.

## Electronic supplementary material

Supplementary Information for Doping-induced carrier profiles in organic semiconductors determined from capacitive extraction-current transients

## References

[1] Siebert-Henze E (2014). Electroabsorption studies of organic p-i-n solar cells: Increase of the built-in voltage by higher doping concentration in the hole transport layer. Org. Electron.

[2] Kim YH, Schubert S, Timmreck R, Müller-Meskamp L, Leo K (2013). Collecting the electrons on n-doped fullerene C_60_ transparent conductors for all-vacuum-deposited small-molecule organic solar cells. Adv. Energy Mater..

[3] Günther AA, Sawatzki M, Formánek P, Kasemann D, Leo K (2016). Contact doping for vertical organic field-effect transistors. Adv. Funct. Mater..

[4] Guillain F (2016). Solution-processed p-dopant as interlayer in polymer solar cells. ACS Appl. Mater. Interfaces.

[5] Dai A (2014). Enhanced charge-carrier injection and collection via lamination of doped polymer layers p-doped with a solution-processible molybdenum complex. Adv. Funct. Mater..

[6] Tress W, Corvers S, Leo K, Riede M (2013). Investigation of driving forces for charge extraction in organic solar cells: transient photocurrent measurements on solar cells showing s-shaped current–voltage characteristics. Adv. Energy Mater..

[7] Sandberg OJ, Nyman M, Österbacka R (2014). The effect of contacts in bulk heterojunction solar cells. Phys. Rev. Applied.

[8] Sandberg OJ, Sundqvist A, Nyman M, Österbacka R (2016). Relating charge transport, contact properties and recombination to open-circuit voltage in sandwich-type thin-film solar cells. Phys. Rev. Applied.

[9] Lüssem B, Riede M, Leo K (2013). Doping of organic semiconductors. Phys. Status Solidi A.

[10] Salzmann I (2012). Intermolecular hybridization governs molecular electrical doping. Phys. Rev. Lett..

[11] Mityashin A (2012). Unraveling the mechanism of molecular doping in organic semiconductors. Adv. Mater..

[12] Tietze ML, Burtone L, Riede M, Lüssem B, Leo K (2012). Fermi level shift and doping efficiency in p-doped small molecule organic semiconductors: A photoelectron spectroscopy and theoretical study. Phys. Rev. B.

[13] Dai A (2015). Investigation of p-dopant diffusion in polymer films and bulk heterojunctions: Stable spatially-confined doping for all-solution processed solar cells. Org. Electron..

[14] Nyman M, Dahlström S, Sandberg OJ, Österbacka R (2016). Unintentional bulk doping of polymer-fullerene blends from a thin interfacial layer of MoO_3_. Adv. Energy Mater.

[15] Seemann A (2011). Reversible and irreversible degradation of organic solar cell performance by oxygen. Solar Energy.

[16] Mateker WR (2013). Improving the long-term stability of PBDTTPD polymer solar cells through material purification aimed at removing organic impurities. Energy Environ. Sci..

[17] Tietze ML, Leo K, Lüssem B (2013). Quantification of deep hole-trap filling by molecular p-doping: Dependence on the host material purity. Org. Electron..

[18] Olthof S, Tress W, Meerheim R, Lüssem B, Leo K (2009). Photoelectron spectroscopy study of systematically varied doping concentrations in an organic semiconductor layer using a molecular p-dopant. J. Appl. Phys..

[19] Pahner P (2013). Pentacene Schottky diodes studied by impedance spectroscopy: Doping properties and trap response. Phys. Rev. B.

[20] Menke T, Ray D, Kleemann H, Leo K, Riede M (2015). Determining doping efficiency and mobility from conductivity and Seebeck data of n-doped C_60_ layers. Phys. Status Solidi B.

[21] Menke T (2014). Highly efficient p-dopants in amorphous hosts. Org. Electron..

[22] Sandberg OJ, Nyman M, Österbacka R (2014). Direct determination of doping concentration and built-in voltage from extraction current transients. Org. Electron..

[23] Sundqvist A, Sandberg OJ, Nyman M, Smått J-H, Österbacka R (2016). Origin of the s-shaped JV curve and the light-soaking issue in inverted organic solar cells. Adv. Energy Mater..

[24] Lampert, M. A., Mark, P. *Current Injection in Solids* (Academic Press, New York, 1970).

[25] Sze, S. M. *Physics of Semiconductor Devices* (Wiley & Sons, New York, 1981).

[26] Johnson WC, Panousis PT (1971). The influence of Debye length on the C-V measurement of doping profiles. IEEE Trans. Electron Devices ED.

[27] Kroemer H, Chien W-Y (1981). On the theory of Debye averaging in the C-V profiling of semiconductors. Solid-State Electronics.

[28] Murawski C, Fuchs C, Hofmann S, Leo K, Gather MC (2014). Alternative p-doped hole transport material for low operating voltage and high efficiency organic light-emitting diodes. Appl. Phys. Lett..

