# Supplementary material for: Doping-induced carrier profiles in organic semiconductors determined from capacitive extraction-current transients
Source: Sci Rep. 2017 Jul 14;7:5397. doi: 10.1038/s41598-017-05499-3 (PMC5511276; doi:10.1038/s41598-017-05499-3)
Supplement: Supplementary file 1 — Supplementary Information for Doping-induced carrier profiles in organic semiconductors determined from capacitive extraction-current transients [file 41598_2017_5499_MOESM1_ESM.pdf]

Supplementary Information for

Doping-induced carrier profiles in organic semiconductors determined from capacitive extraction-current transients

Mathias Nyman[1], Oskar J. Sandberg[1], Staffan Dahlström[1], Donato Spoltore[2], Christian Körner[2], Yadong Zhang [3], Stephen Barlow[3], Seth R. Marder[3], Karl Leo[2], Koen Vandewal[2], and Ronald Österbacka[1]

[1]Physics/Faculty of Science and Engineering, and Center for Functional Materials, Åbo Akademi University, Porthansgatan 3, 20500 Turku, Finland

[2]Dresden Integrated Center for Applied Physics and Photonic Materials (IAPP) and Institute für Angewandte Physik, Technische Universität Dresden, Nöthnitzer Straße 61, 01187 Dresden, Germany

[3]School of Chemistry and Biochemistry and Center for Organic Photonics and Electronics, Georgia Institute of Technology, Atlanta, Georgia 30332-0400, United States

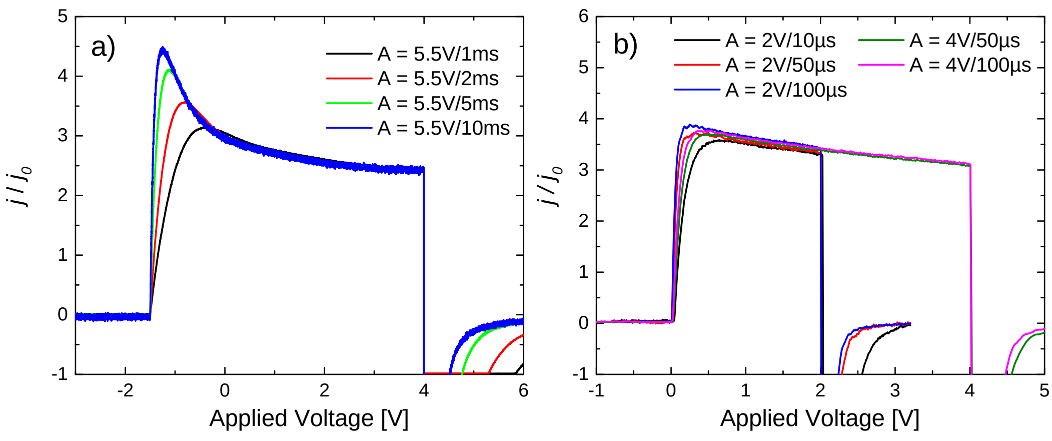

Figure S1. Extraction current transients with varying voltage slopes *A* for a) a BF-DPB:$C_{60}F_{36}$ devices with two layers of different dopant concentrations; in the lower doped layer (d = 40 nm) the $C_{60}F_{36}$ to BF-DPB weight ratio is 0.2 % and 4 wt% in the more heavily doped layer (d = 70 nm) b) a doped ITO/P3HT/Al device with 0.2 wt % dopants.

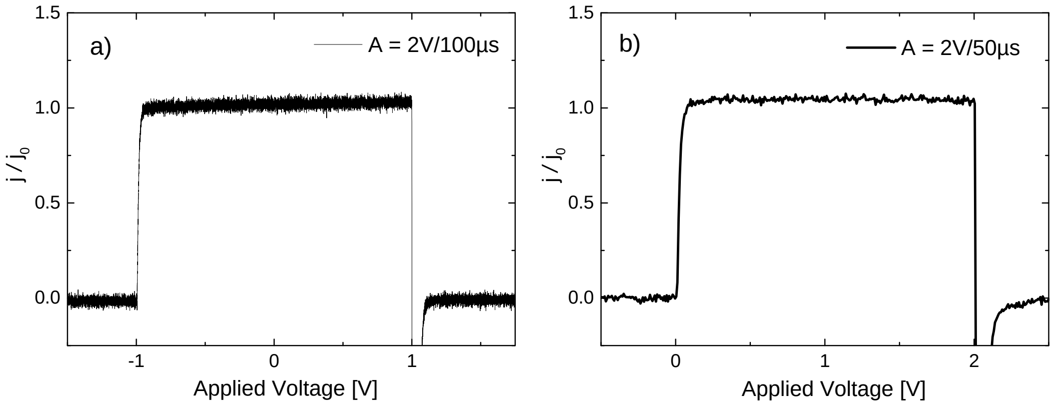

Figure S2. Extraction current transients of a) an undoped of ITO/BF-DPB/LiF/Al device and b) an undoped ITO/P3HT/Al device. Only the geometric capacitive response, j_0, is seen since no free charges are present in the undoped active layer.
